# 2-(2-Methyl­benzo­yl)benzoic acid: catemeric hydrogen bonding in a γ-keto acid^1^

**DOI:** 10.1107/S1600536813025099

**Published:** 2013-09-18

**Authors:** Natalia A. Platosz, Roger A. Lalancette, Hugh W. Thompson, Jacob M. Newman, Ari Schachter

**Affiliations:** a Carl A. Olson Memorial Laboratories, Department of Chemistry, Rutgers University, Newark, NJ 07102 , USA; b Department of Chemistry, Touro College - Lander College for Men, New York, NY , USA

## Abstract

The crystal structure of the title compound, C_15_H_12_O_3_, displays catemeric aggregation involving O—H⋯O hydrogen bonds progressing from the carboxyl group of one mol­ecule to the ketone O atom of another glide-related neighbor. The mol­ecule is twisted, with the toluene 80.61 (3)° out of plane with respect to the phenyl group of the benzoic acid. The acid group makes a dihedral angle of 13.79 (14)° with the attached phenyl ring. The mol­ecules are achiral, but the space group glide planes create alternating conformational chirality in the chain units. The four hydrogen-bonding chains progress along [001] in an *A*—*A*—*B*—*B* pattern (right-to-left *versus* left-to-right), and are related to each other by the center of symmetry at (0.5, 0.5, 0.5) in the chosen cell. There is one close contact (2.54 Å) between a phenyl H atom and the acid carbonyl from a symmetry-related mol­ecule.

## Related literature

For a discussion of highly ordered carboxyl bond distances and angles, see: Borthwick (1980 ▶). For close contact information, see: Steiner (1997 ▶). For related structures, see: Abell *et al.* (1991 ▶); Barcon *et al.* (1998 ▶, 2002 ▶); Degen & Bolte (1999 ▶); Hickmott *et al.* (1985 ▶); Kashyap *et al.* (1995 ▶); Song *et al.* (2008 ▶); Thompson *et al.* (1998 ▶); Watson *et al.* (1990 ▶). For preparation of the title compound, see: Newman & McCleary (1941 ▶). For a description of the Cambridge Structural Database, see: Allen (2002 ▶).
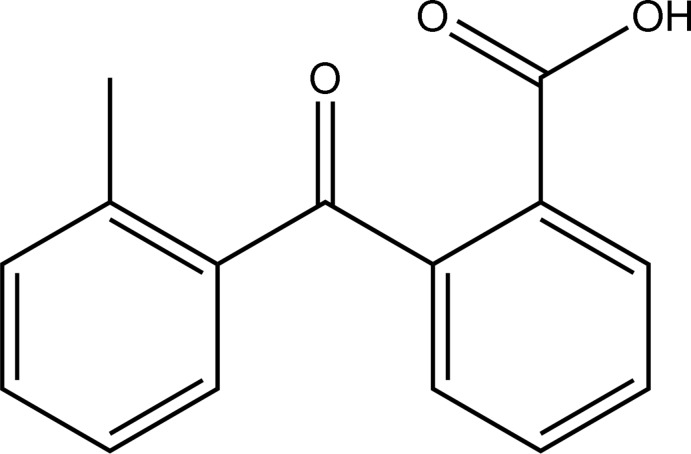


## Experimental

### 

#### Crystal data

C_15_H_12_O_3_
*M* *_r_* = 240.25
Orthorhombic, 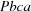

*a* = 10.7450 (2) Å
*b* = 10.1737 (2) Å
*c* = 21.6588 (4) Å
*V* = 2367.66 (8) Å^3^
*Z* = 8
Cu *K*α radiation
μ = 0.77 mm^−1^
*T* = 100 K
0.45 × 0.26 × 0.20 mm

#### Data collection

Bruker SMART CCD APEXII area-detector diffractometer
Absorption correction: numerical (*SADABS*; Sheldrick, 2008*a* ▶) *T*_min_ = 0.724, *T*_max_ = 0.862
20799 measured reflections
2143 independent reflections
2075 reflections with *I* > 2σ(*I*)
*R*_int_ = 0.024

#### Refinement

*R*[*F* ^2^ > 2σ(*F* ^2^)] = 0.030
*wR*(*F* ^2^) = 0.076
*S* = 1.07
2143 reflections
169 parameters
H atoms treated by a mixture of independent and constrained refinement
Δρ_max_ = 0.24 e Å^−3^
Δρ_min_ = −0.17 e Å^−3^

### 

Data collection: *APEX2* (Bruker, 2006 ▶); cell refinement: *SAINT* (Bruker, 2005 ▶); data reduction: *SAINT*; program(s) used to solve structure: *SHELXTL* (Sheldrick, 2008*b* ▶); program(s) used to refine structure: *SHELXTL*; molecular graphics: *SHELXTL*; software used to prepare material for publication: *SHELXTL*.

## Supplementary Material

Crystal structure: contains datablock(s) I, global. DOI: 10.1107/S1600536813025099/bg2513sup1.cif

Structure factors: contains datablock(s) I. DOI: 10.1107/S1600536813025099/bg2513Isup2.hkl

Supplementary material file. DOI: 10.1107/S1600536813025099/bg2513Isup3.cml

Additional supplementary materials:  crystallographic information; 3D view; checkCIF report

## Figures and Tables

**Table 1:** Hydrogen-bond geometry (Å, °)

*D*—H⋯*A*	*D*—H	H⋯*A*	*D*⋯*A*	*D*—H⋯*A*
O3—H3*A*⋯O1^ii^	0.932 (18)	1.714 (18)	2.6477 (11)	172.3 (16)

Symmetry code: (ii) 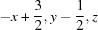.

**Fig. 1.**
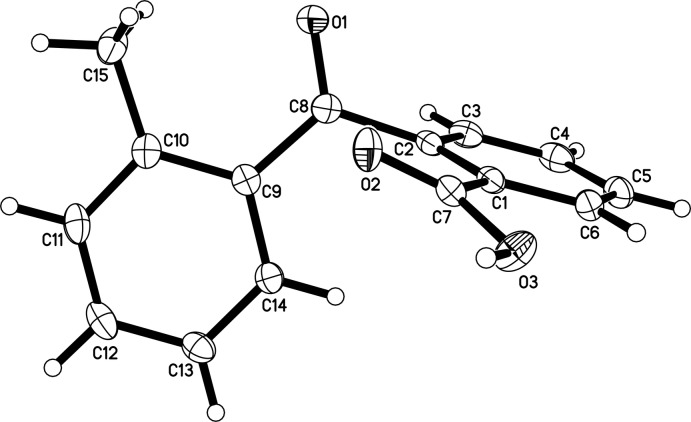
A view of the asymmetric unit of (I) with its numbering scheme. Displacement ellipsoids are drawn at a 40% probability level for non-H atoms.

**Fig. 2.**
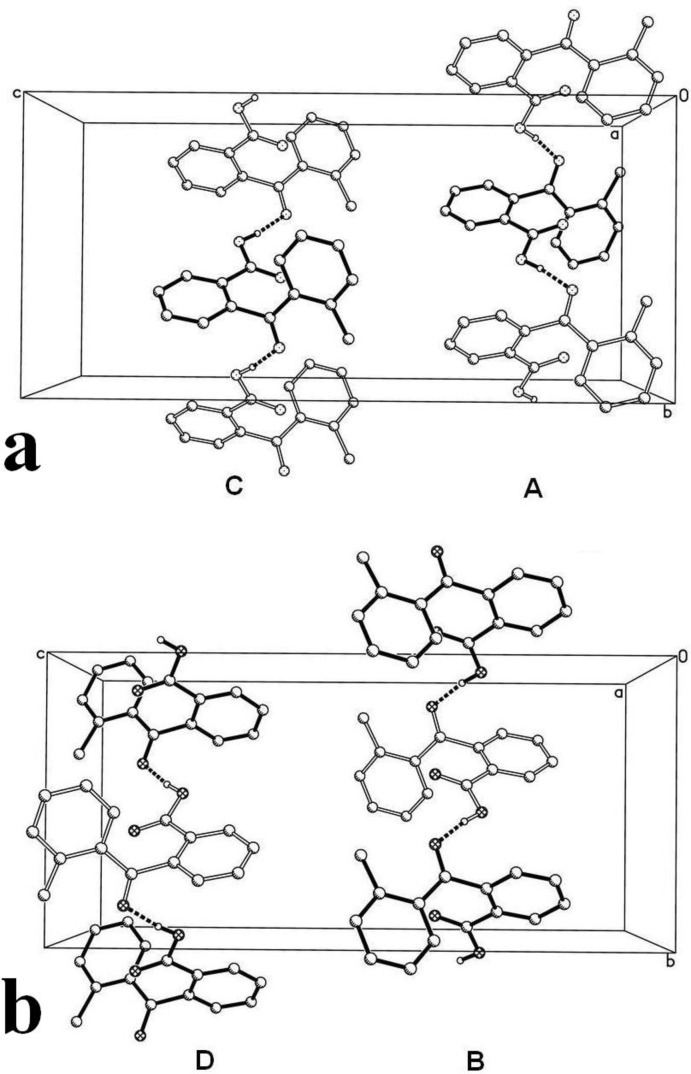
Partial packing diagrams for (I). In each part, two different chains of glide-related H-bonding molecules are shown, connected by O—H···O hydrogen bonds (dashed lines). The handedness of the molecules alternates along each individual chain, and is shown by alternate shading of the bonds. The chain marked A has its centrosymmetric counterpart labeled D in the 2nd figure. The chain labeled B (with opposite H-bonding direction to A) has its centrosymmetric counterpart shown as C generated through the center of symmetry. H atoms not involved in hydrogen bonding have been omitted for clarity.

## supplementary crystallographic information

### 1. Comment

This carboxyl-to-ketone catemer (I) is an achiral molecule which generates heterochiral chains due to the glide-related chain members. Normally, these types of catemers involve homochiral chains having either a screw or translational internal relationship (Hickmott *et al.*, 1985; Abell *et al.*, 1991; Kashyap *et al.*, 1995). Rarer are heterochiral chains resulting from glide-plane symmetry, which is the case here (Watson *et al.*, 1990; Barcon *et al.*, 1998, 2002; Thompson *et al.*, 1998).

Fig. 1 shows the asymmetric unit for (I) with its numbering. The molecule has many options for rotation, allowing for the angle between the toluene ring and the phenyl ring of the benzoic acid to be nearly perpendicular at 80.61 (3)°. The acid group [C7—O2—O3] makes a dihedral angle with the phenyl group [C1—C6] = 13.79 (14)°, and the ketone [O1—C8—C2—C9] has a dihedral angle to the phenyl group [C1—C6] = 78.99 (3)°. The acid group [C7—O2—O3] makes a dihedral angle of 74.84 (8)° with the plane of the ketone [O1—C8—C2—C9], and also an angle of 76.65 (7)° with the plane of the toluene ring [C9—C14]. There is one close contact (2.54 Å) between a phenyl H atom (H12) and the acid carbonyl (O2) from a symmetry-related molecule (Steiner, 1997).

The structures of 2-(4-methylbenzoyl)benzoic acid and its hydrate have been published: HOFGAK (Degen & Bolte, 1999) is the *p*-toluene analog of (I) and MIXTOD (Song, *et al.*, 2008) is the hydrated *p*-toluene analog [Cambridge Structural Database (CSD, Version 5.28, update of Nov., 2006; Allen, 2002)]. Both of these molecules crystallize as centrosymmetric dimers of the acid groups (Borthwick, 1980), but the hydrated version has two water molecules inserted between the acid dimers. In HOFGAK, the dihedral angle between the toluene and the benzoic acid = 89.8°, and the acid is coplanar [0.00°] with the phenyl ring. In MIXTOD, these same angles are 69.5 and 25.8°, respectively. In (I), the toluene and the ketone are essentially coplanar [dihedral angle = 1.91 (7)°], but in HOFGAK and MIXTOD, this same angle is 20.6 and 10.8°, respectively.

Figures 2a and 2b are partial packing diagrams showing the H bonds progressing catemerically between glide-related molecules; the individual members making up each chain have alternating chirality. The handedness of the molecules is differentiated by patterning of the bonds.

Centrosymmetrically-related H-bonded chains can be seen by comparing the A molecule in Fig. 2a with the D molecule in Fig. 2b; the B and C molecules are also centrosymmetrically related.

We characterize the geometry of H bonding to carbonyls using a combination of H···O=C angle and H···O=C—C torsion angle. These describe the approach of the acid H atom to the carbonyl O in terms of its deviation from, respectively, C=O axiality (ideal = 120°) and planarity with the carbonyl (ideal = 0°). In (I), the values for these two angles are 116.5 (5) and 10.0 (6)°.

### 2. Experimental

1.20 g of Mg and 1 ml of 1-bromo-2-methylbenzene were added to 17 ml of dry diethyl ether, followed by the gradual addition of 4.625 ml of 1-bromo-2-methylbenzene. The resulting Grignard reagent was added over a period of 30 min to a stirred suspension of 6.2 g of phthalic anhydride in 35 ml of dry benzene and 15 ml of dry diethyl ether. The pale orange mixture was stirred for 30 minutes, refluxed for 10 minutes, allowed to stir overnight and refluxed for a further 10 minutes. 7 ml of HCl was added along with a small quantity of ice water. The resulting solution was distilled to dryness. The glassy product was then dissolved in aqueous KOH and filtered through Celite. The filtrate was then acidified with HCl and extracted with ether into several fractions. The fractions not yielding crystals melting between 100–110°C were discarded, and the remaining fractions were combined. These were then passed through an alumina column and the solvent allowed to concentrate, yielding pale yellow crystals. These were then recrystallized from 8.6 ml of methanol and 5 mL of water. The resulting fine needle-like crystals were then recrystallized again from pure ethanol at room temperature, yielding colourless block crystals. (See Newman & McCleary, 1941).

### 3. Refinement

Crystal data, data collection and structure refinement details are summarized in Table 1. All H atoms for (I) were found in electron density difference maps. The hydroxyl H was refined. The methyl H atoms were put in ideally staggered positions with C—H distances of 0.98 Å and *U*_iso_(H) = 1.5*U*_eq_(C). The aromatic Hs were placed in geometrically idealized positions and constrained to ride on their parent C atoms with C—H distance of 0.95 Å and *U*_iso_(H) = 1.2*U*_eq_(C).

### Crystal data

**Table:**

C_15_H_12_O_3_	*F*(000) = 1008
*M**_r_* = 240.25	*D*_x_ = 1.348 Mg m^−^^3^
Orthorhombic, *P**b**c**a*	Cu *K*α radiation, λ = 1.54178 Å
Hall symbol: -P 2ac 2ab	Cell parameters from 9881 reflections
*a* = 10.7450 (2) Å	θ = 4.1–69.4°
*b* = 10.1737 (2) Å	µ = 0.77 mm^−^^1^
*c* = 21.6588 (4) Å	*T* = 100 K
*V* = 2367.66 (8) Å^3^	Block, colourless
*Z* = 8	0.45 × 0.26 × 0.20 mm

### Data collection

**Table:**

Bruker SMART CCD APEXII area-detector diffractometer	2143 independent reflections
Radiation source: fine-focus sealed tube	2075 reflections with *I* > 2σ(*I*)
Graphite monochromator	*R*_int_ = 0.024
φ and ω scans	θ_max_ = 69.7°, θ_min_ = 4.1°
Absorption correction: numerical (*SADABS*; Sheldrick, 2008*a*)	*h* = −12→12
*T*_min_ = 0.724, *T*_max_ = 0.862	*k* = −12→11
20799 measured reflections	*l* = −26→25

### Refinement

**Table:**

Refinement on *F*^2^	Secondary atom site location: difference Fourier map
Least-squares matrix: full	Hydrogen site location: inferred from neighbouring sites
*R*[*F*^2^ > 2σ(*F*^2^)] = 0.030	H atoms treated by a mixture of independent and constrained refinement
*wR*(*F*^2^) = 0.076	*w* = 1/[σ^2^(*F*_o_^2^) + (0.0323*P*)^2^ + 0.9262*P*] where *P* = (*F*_o_^2^ + 2*F*_c_^2^)/3
*S* = 1.07	(Δ/σ)_max_ < 0.001
2143 reflections	Δρ_max_ = 0.24 e Å^−^^3^
169 parameters	Δρ_min_ = −0.17 e Å^−^^3^
0 restraints	Extinction correction: *SHELXTL* (Sheldrick, 2008b), Fc^*^=kFc[1+0.001xFc^2^λ^3^/sin(2θ)]^-1/4^
Primary atom site location: structure-invariant direct methods	Extinction coefficient: 0.00145 (14)

### Special details

**Table:**

Experimental. 'crystal mounted on a Cryoloop using Paratone-N'
Geometry. All e.s.d.'s (except the e.s.d. in the dihedral angle between two l.s. planes) are estimated using the full covariance matrix. The cell e.s.d.'s are taken into account individually in the estimation of e.s.d.'s in distances, angles and torsion angles; correlations between e.s.d.'s in cell parameters are only used when they are defined by crystal symmetry. An approximate (isotropic) treatment of cell e.s.d.'s is used for estimating e.s.d.'s involving l.s. planes.
Refinement. Refinement of *F*^2^ against ALL reflections. The weighted *R*-factor *wR* and goodness of fit *S* are based on *F*^2^, conventional *R*-factors *R* are based on *F*, with *F* set to zero for negative *F*^2^. The threshold expression of *F*^2^ > σ(*F*^2^) is used only for calculating *R*-factors(gt) *etc*. and is not relevant to the choice of reflections for refinement. *R*-factors based on *F*^2^ are statistically about twice as large as those based on *F*, and *R*- factors based on ALL data will be even larger.

### Fractional atomic coordinates and isotropic or equivalent isotropic displacement parameters (Å^2^)

**Table:**

	*x*	*y*	*z*	*U*_iso_*/*U*_eq_	
O1	0.94221 (8)	0.35848 (8)	0.62029 (4)	0.0310 (2)	
O2	0.77379 (7)	0.10436 (8)	0.62358 (3)	0.0265 (2)	
O3	0.71268 (8)	−0.03026 (8)	0.69973 (4)	0.0296 (2)	
H3A	0.6595 (17)	−0.0633 (17)	0.6695 (8)	0.059 (5)*	
C1	0.86743 (10)	0.12547 (10)	0.72243 (5)	0.0192 (2)	
C2	0.96175 (10)	0.20965 (10)	0.70233 (5)	0.0194 (2)	
C3	1.04111 (10)	0.26693 (11)	0.74557 (5)	0.0247 (3)	
H3	1.1060	0.3236	0.7322	0.030*	
C4	1.02588 (11)	0.24164 (12)	0.80801 (5)	0.0285 (3)	
H4	1.0800	0.2815	0.8372	0.034*	
C5	0.93210 (11)	0.15848 (12)	0.82799 (5)	0.0282 (3)	
H5	0.9218	0.1413	0.8708	0.034*	
C6	0.85359 (11)	0.10055 (11)	0.78538 (5)	0.0239 (3)	
H6	0.7896	0.0432	0.7991	0.029*	
C7	0.78069 (10)	0.06640 (10)	0.67642 (5)	0.0205 (2)	
C8	0.97910 (10)	0.24909 (10)	0.63557 (5)	0.0209 (2)	
C9	1.04899 (9)	0.16186 (10)	0.59332 (5)	0.0197 (2)	
C10	1.07236 (10)	0.19649 (11)	0.53104 (5)	0.0220 (2)	
C11	1.14050 (10)	0.10824 (12)	0.49536 (5)	0.0272 (3)	
H11	1.1585	0.1303	0.4537	0.033*	
C12	1.18287 (11)	−0.01046 (12)	0.51844 (5)	0.0282 (3)	
H12	1.2278	−0.0689	0.4925	0.034*	
C13	1.15975 (11)	−0.04392 (11)	0.57930 (5)	0.0257 (3)	
H13	1.1892	−0.1249	0.5955	0.031*	
C14	1.09334 (10)	0.04195 (10)	0.61622 (5)	0.0217 (2)	
H14	1.0775	0.0191	0.6580	0.026*	
C15	1.02565 (11)	0.32089 (12)	0.50152 (5)	0.0286 (3)	
H15A	1.0573	0.3267	0.4592	0.043*	
H15B	1.0548	0.3968	0.5253	0.043*	
H15C	0.9345	0.3201	0.5009	0.043*	

### Atomic displacement parameters (Å^2^)

**Table:**

	*U*^11^	*U*^22^	*U*^33^	*U*^12^	*U*^13^	*U*^23^
O1	0.0390 (5)	0.0237 (4)	0.0303 (4)	0.0084 (4)	0.0099 (4)	0.0048 (3)
O2	0.0248 (4)	0.0380 (5)	0.0166 (4)	−0.0045 (3)	0.0004 (3)	−0.0019 (3)
O3	0.0312 (5)	0.0272 (4)	0.0303 (4)	−0.0101 (3)	−0.0078 (4)	0.0056 (3)
C1	0.0196 (5)	0.0190 (5)	0.0191 (5)	0.0039 (4)	0.0003 (4)	−0.0028 (4)
C2	0.0198 (5)	0.0174 (5)	0.0210 (5)	0.0046 (4)	0.0006 (4)	−0.0035 (4)
C3	0.0224 (5)	0.0204 (5)	0.0312 (6)	0.0011 (4)	−0.0030 (5)	−0.0047 (5)
C4	0.0319 (6)	0.0269 (6)	0.0268 (6)	0.0063 (5)	−0.0105 (5)	−0.0098 (5)
C5	0.0365 (7)	0.0308 (6)	0.0172 (5)	0.0085 (5)	−0.0024 (5)	−0.0038 (5)
C6	0.0260 (6)	0.0251 (6)	0.0206 (5)	0.0037 (5)	0.0029 (4)	−0.0004 (4)
C7	0.0195 (5)	0.0208 (5)	0.0211 (5)	0.0020 (4)	0.0023 (4)	−0.0030 (4)
C8	0.0177 (5)	0.0207 (5)	0.0243 (6)	−0.0020 (4)	0.0003 (4)	0.0001 (4)
C9	0.0154 (5)	0.0227 (5)	0.0210 (5)	−0.0029 (4)	−0.0008 (4)	−0.0026 (4)
C10	0.0164 (5)	0.0292 (6)	0.0204 (5)	−0.0038 (4)	−0.0026 (4)	−0.0013 (4)
C11	0.0223 (6)	0.0423 (7)	0.0169 (5)	−0.0007 (5)	−0.0008 (4)	−0.0042 (5)
C12	0.0209 (6)	0.0385 (6)	0.0254 (6)	0.0049 (5)	−0.0011 (5)	−0.0120 (5)
C13	0.0229 (6)	0.0260 (6)	0.0281 (6)	0.0042 (4)	−0.0031 (5)	−0.0050 (5)
C14	0.0204 (5)	0.0242 (5)	0.0205 (5)	−0.0012 (4)	−0.0007 (4)	−0.0021 (4)
C15	0.0271 (6)	0.0366 (6)	0.0222 (5)	0.0002 (5)	−0.0001 (5)	0.0053 (5)

### Geometric parameters (Å, º)

**Table:**

O1—C8	1.2269 (13)	C8—C9	1.4796 (15)
O2—C7	1.2102 (13)	C9—C14	1.4004 (15)
O3—C7	1.3251 (13)	C9—C10	1.4165 (15)
O3—H3A	0.932 (18)	C10—C11	1.3926 (16)
C1—C6	1.3948 (15)	C10—C15	1.5041 (16)
C1—C2	1.3965 (15)	C11—C12	1.3840 (18)
C1—C7	1.4909 (15)	C11—H11	0.9500
C2—C3	1.3942 (15)	C12—C13	1.3838 (17)
C2—C8	1.5120 (15)	C12—H12	0.9500
C3—C4	1.3863 (17)	C13—C14	1.3828 (15)
C3—H3	0.9500	C13—H13	0.9500
C4—C5	1.3851 (18)	C14—H14	0.9500
C4—H4	0.9500	C15—H15A	0.9800
C5—C6	1.3823 (16)	C15—H15B	0.9800
C5—H5	0.9500	C15—H15C	0.9800
C6—H6	0.9500		
			
H12···O2^i^	2.54		
			
C7—O3—H3A	109.8 (11)	C14—C9—C10	119.58 (10)
C6—C1—C2	119.57 (10)	C14—C9—C8	118.43 (9)
C6—C1—C7	120.89 (10)	C10—C9—C8	121.99 (10)
C2—C1—C7	119.51 (9)	C11—C10—C9	117.47 (10)
C3—C2—C1	119.40 (10)	C11—C10—C15	118.82 (10)
C3—C2—C8	117.13 (10)	C9—C10—C15	123.70 (10)
C1—C2—C8	123.38 (9)	C12—C11—C10	122.35 (10)
C4—C3—C2	120.37 (11)	C12—C11—H11	118.8
C4—C3—H3	119.8	C10—C11—H11	118.8
C2—C3—H3	119.8	C13—C12—C11	119.98 (11)
C5—C4—C3	120.26 (11)	C13—C12—H12	120.0
C5—C4—H4	119.9	C11—C12—H12	120.0
C3—C4—H4	119.9	C14—C13—C12	119.21 (11)
C6—C5—C4	119.73 (11)	C14—C13—H13	120.4
C6—C5—H5	120.1	C12—C13—H13	120.4
C4—C5—H5	120.1	C13—C14—C9	121.41 (10)
C5—C6—C1	120.66 (11)	C13—C14—H14	119.3
C5—C6—H6	119.7	C9—C14—H14	119.3
C1—C6—H6	119.7	C10—C15—H15A	109.5
O2—C7—O3	124.28 (10)	C10—C15—H15B	109.5
O2—C7—C1	122.80 (10)	H15A—C15—H15B	109.5
O3—C7—C1	112.91 (9)	C10—C15—H15C	109.5
O1—C8—C9	122.77 (10)	H15A—C15—H15C	109.5
O1—C8—C2	117.31 (9)	H15B—C15—H15C	109.5
C9—C8—C2	119.67 (9)		
			
C6—C1—C2—C3	0.35 (15)	C3—C2—C8—C9	99.68 (11)
C7—C1—C2—C3	178.67 (9)	C1—C2—C8—C9	−83.75 (13)
C6—C1—C2—C8	−176.14 (10)	O1—C8—C9—C14	176.35 (10)
C7—C1—C2—C8	2.17 (15)	C2—C8—C9—C14	2.36 (14)
C1—C2—C3—C4	−0.62 (16)	O1—C8—C9—C10	−3.38 (16)
C8—C2—C3—C4	176.09 (10)	C2—C8—C9—C10	−177.37 (9)
C2—C3—C4—C5	0.43 (17)	C14—C9—C10—C11	−0.57 (15)
C3—C4—C5—C6	0.04 (17)	C8—C9—C10—C11	179.15 (10)
C4—C5—C6—C1	−0.31 (17)	C14—C9—C10—C15	178.13 (10)
C2—C1—C6—C5	0.11 (16)	C8—C9—C10—C15	−2.14 (16)
C7—C1—C6—C5	−178.18 (10)	C9—C10—C11—C12	1.15 (16)
C6—C1—C7—O2	165.34 (10)	C15—C10—C11—C12	−177.62 (11)
C2—C1—C7—O2	−12.94 (15)	C10—C11—C12—C13	−1.14 (18)
C6—C1—C7—O3	−14.30 (14)	C11—C12—C13—C14	0.51 (17)
C2—C1—C7—O3	167.41 (9)	C12—C13—C14—C9	0.05 (17)
C3—C2—C8—O1	−74.63 (13)	C10—C9—C14—C13	0.00 (16)
C1—C2—C8—O1	101.94 (12)	C8—C9—C14—C13	−179.74 (10)

Symmetry code: (i) −*x*+2, −*y*, −*z*+1.

### Hydrogen-bond geometry (Å, º)

**Table:**

*D*—H···*A*	*D*—H	H···*A*	*D*···*A*	*D*—H···*A*
O3—H3*A*···O1^ii^	0.932 (18)	1.714 (18)	2.6477 (11)	172.3 (16)

Symmetry code: (ii) −*x*+3/2, *y*−1/2, *z*.

## References

[bb1] Abell, A. D., Trent, J. & Robinson, W. P. (1991). *J. Chem. Soc. Chem. Commun.* pp. 362–363.

[bb2] Allen, F. H. (2002). *Acta Cryst.* B**58**, 380–388. 10.1107/s0108768102003890 12037359

[bb3] Barcon, A., Brunskill, A. P. J., Lalancette, R. A. & Thompson, H. W. (1998). *Acta Cryst.* C**54**, 1282–1285.

[bb4] Barcon, A., Brunskill, A. P. J., Lalancette, R. A. & Thompson, H. W. (2002). *Acta Cryst.* C**58**, o154–o156. 10.1107/s0108270101020595 11870311

[bb5] Borthwick, P. W. (1980). *Acta Cryst.* B**36**, 628–632.

[bb6] Bruker (2005). *SAINT* Bruker AXS Inc., Madison, Wisconsin, USA.

[bb7] Bruker (2006). *APEX2* Bruker AXS Inc., Madison, Wisconsin, USA.

[bb8] Degen, A. & Bolte, M. (1999). *Acta Cryst.* C**55**, 1306–1308.

[bb9] Hickmott, P. W., Ahmed, M. G., Ahmed, S. A., Wood, S. & Kapon, M. (1985). *J. Chem. Soc. Perkin Trans. 1*, pp. 2559–2571.

[bb10] Kashyap, R. P., Deshpande, M. N., Rajapaksa, D., Marchand, A. P. & Watson, W. H. (1995). *J. Chem. Crystallogr.* **25**, 573–578.

[bb11] Newman, M. S. & McCleary, C. D. (1941). *J. Am. Chem. Soc.* **63**, 1537–1541.

[bb12] Sheldrick, G. M. (2008*a*). *SADABS* University of Göttingen, Germany.

[bb13] Sheldrick, G. M. (2008*b*). *Acta Cryst.* A**64**, 112–122. 10.1107/S0108767307043930 18156677

[bb14] Song, G.-L., Deng, S.-P., Liu, S. & Zhu, H.-J. (2008). *Acta Cryst.* E**64**, o894. 10.1107/S1600536808010581 PMC2961241 21202377

[bb15] Steiner, T. (1997). *Chem. Commun.* pp. 727–734.

[bb16] Thompson, H. W., Brunskill, A. P. J. & Lalancette, R. A. (1998). *Acta Cryst.* C**54**, 829–831.

[bb17] Watson, W. H., Nagl, A., Kashyap, R. P., Marchand, A. P. & Vidyasagar, V. (1990). *Acta Cryst.* C**46**, 1265–1268.

